# Intranasal administration enhances size-dependent pulmonary phagocytic uptake of poly(lactic-*co*-glycolic acid) nanoparticles

**DOI:** 10.1186/s41181-023-00227-x

**Published:** 2024-02-15

**Authors:** Seung Ho Baek, Eun-Ha Hwang, Gyeung Haeng Hur, Green Kim, You Jung An, Jae-Hak Park, Jung Joo Hong

**Affiliations:** 1 National Primate Research Centre, Korea Research Institute of Bioscience and Biotechnology (KRIBB), Yeongudanji-ro, Ochang-eup, Chengwon-gu, Cheongju, Chungcheongbuk 28116 Republic of Korea; 2 KRIBB School of Bioscience, Korea University of Science and Technology (UST), Daejeon, Republic of Korea; 3 Department of Laboratory Animal Medicine, College of Veterinary Medicine, Seoul National University, Gwanak-gu, Seoul, 08826 Republic of Korea; 4 Agency for Defense Development, Daejeon, Republic of Korea

**Keywords:** Pulmonary delivery, PLGA nanoparticle, Intranasal administration, Nanocarrier

## Abstract

**Background:**

Nanoparticles exhibit distinct behaviours within the body, depending on their physicochemical properties and administration routes. However, in vivo behaviour of poly(lactic-*co*-glycolic acid) (PLGA) nanoparticles, especially when administered nasally, remains unexplored; furthermore, there is a lack of comparative analysis of uptake efficiency among different administration routes. Therefore, here, we aimed to comprehensively investigate the real-time in vivo behaviour of PLGA nanoparticles across various administration routes. PLGA-NH_2_ nanoparticles of three sizes were synthesised using an oil-in-water single-emulsion method. We assessed their uptake by murine macrophage RAW264.7 cells using fluorescence microscopy. To enable real-time tracking, we conjugated p-SCN-Bn-deferoxamine to PLGA-NH_2_ nanoparticles and further radiolabelled them with ^89^Zr-oxalate before administration to mice via different routes. Nanoparticle internalisation by lung immune cells was monitored using fluorescence-activated cell sorting analysis.

**Results:**

The nanoparticle sizes were 294 ± 2.1 (small), 522.5 ± 5.58 (intermediate), and 850 ± 18.52 nm (large). Fluorescent labelling did not significantly alter the nanoparticle size and charge. The level of uptake of small and large nanoparticles by RAW264.7 cells was similar, with phagocytosis inhibition primarily reducing the internalisation of large particles. Positron emission tomography revealed that intranasal delivery resulted in the highest and most targeted pulmonary uptake, whereas intravenous administration led to accumulation mainly in the liver and spleen. Nasal delivery of large nanoparticles resulted in enhanced uptake by myeloid immune cells relative to lymphoid cells, whereas dendritic cell uptake initially peaked but declined over time.

**Conclusions:**

Our study provides valuable insights into advancing nanomedicine and drug delivery, with the potential for expanding the clinical applications of nanoparticles.

**Supplementary Information:**

The online version contains supplementary material available at 10.1186/s41181-023-00227-x.

## Background

Nanoparticles have the potential to minimise adverse effects and help achieve desired pharmacological outcomes with lower drug amounts by preventing drug degradation and enhancing solubility and bioavailability (Liu et al. 2016; Mitchell et al. 2021; Yao et al. 2020). However, despite their advantages, nanoparticles have certain limitations, including toxicity, challenges in upscaling the manufacturing process owing to complex synthesis processes, and concerns regarding structural stability, warranting further research and development efforts (Mitchell et al. 2021; Hashemzadeh et al. 2020; Zhang et al. 2022). In this context, poly(lactic-*co*-glycolic acid) (PLGA), a biocompatible and biodegradable polymer approved for medical use by both the U.S. Food and Drug Administration and the European Medicines Agency, has garnered substantial attention in the field of drug delivery systems (Operti et al. 2021; Yetisgin et al. 2020), owing to its low toxicity, relatively simple synthesis processes, and satisfactory structural stability, which enables sustained drug release. Consequently, PLGA is a prominent choice in nanomedicine research (Benhabbour et al. 2019).

The physicochemical characteristics of nanoparticles considerably influence their behaviour in the body (Liu et al. 2017; Shi et al. 2021; Wang et al. 2021). Nanoparticles efficiently penetrate desired lung tissues and are readily absorbed into cells. In contrast, lipid nanoparticles demonstrate different outcomes, with micro-sized particles exhibiting longer retention in the lungs than nano-sized particles (Fu et al. 2019; He et al. 2020). However, the lack of comprehensive insights into in vivo nanoparticle behaviour, considering size, shape, surface charge, and chemical modifications, currently hinders the widespread clinical translation of nanoparticles in nanomedicine. The administration method also substantially affects nanoparticle behaviour in vivo, especially tissue absorption, metabolism, and elimination. For instance, delivering nanoparticles via the respiratory tract can circumvent the reticuloendothelial system and hepatic first pass, thereby augmenting lung-specific delivery while reducing systemic adverse effects, especially when compared to conventional intravenous and oral routes (Abdifetah and Na-Bangchang 2019; Nie 2010). Furthermore, the lungs not only provide a large surface area for rapid drug absorption but also exhibit low expression of drug-metabolising enzymes, thereby minimising drug degradation (Rijt et al. 2014). Nevertheless, the intratracheal administration method, involving invasive surgical procedures, might impede the broader clinical applicability of nanoparticle delivery, and there has also been a reported case of non-uniform lung distribution (Wu et al. 2020). In this context, the exploration of nanoparticle administration via the nasal route has recently gained attention; however, previous studies have primarily focused on brain-related applications (Nigam et al. 2019; Sharma et al. 2015) and research on lung-specific nanoparticle delivery through nasal administration remains relatively scarce.

Non-invasive imaging modalities, including fluorescence imaging, magnetic resonance imaging (MRI), and positron emission tomography (PET), are commonly employed to elucidate the in vivo dynamics of nanoparticles. Each of these methods has distinct advantages and limitations. For instance, fluorescence imaging is limited by signal attenuation and elevated background noise, which reduce precision. MRI provides satisfactory spatial resolution and involved the use of non-ionising radiation; however, it may have lower sensitivity, potentially posing challenges in accurately quantifying nanoparticle distribution in minimal quantities of samples. In contrast, PET capitalises on its sensitivity, enabling precise signal acquisition from nanoparticles within tissues. In the context of pulmonary applications, the use of long-half-life radioisotopes in PET allows for the continuous monitoring of nanoparticle behaviour within the respiratory system with a single administration. While intranasal administration present a non-invasive approach with potential clinical applications, its effectiveness is constrained by dosage capacity. The application of highly sensitive PET imaging to intranasal administration not only enables the quantitative analysis of low-dose nanoparticle distribution but also allows for the confirmation of accurate administration. Therefore, PET has emerged as the most suitable analytical modality for predicting the outcome and safety of nanoparticles in major organs.

In this study, we aimed to comprehensively elucidate the real-time in vivo behaviour of PLGA nanoparticles, contingent on the route of administration. Following intranasal administration of PLGA nanoparticles in mice, we examined the uniform dispersion of the nanoparticles within the lungs and compared their uptake efficiency between intranasal and other administration routes (intravenous, intramuscular, and subcutaneous) using PET imaging.

## Materials and Methods

### ***Preparation and characterisation of PLGA-NH***_***2***_*** nanoparticles***

PLGA-NH_2_ nanoparticles (Mw: 20,000; lactic acid:glycolic acid = 50:50; Nanosoft Polymers) were prepared using a modified oil-in-water single-emulsion solvent evaporation method (McCall and Sirianni 2013). Briefly, for nanoparticles with a target size of 300 nm, 60 mg of PLGA-NH_2_ was dissolved in 3 mL of dichloromethane to form an oil phase. This oil phase was added to 15 mL of 0.25% (w/v) polyvinyl alcohol (PVA) solution (Mw: 13–23 kDa; 87–89% hydrolysed) and emulsified for 2 min at 20% amplitude in an ice bath using a probe sonicator (Qsonica 700, Newtown, CT, USA). The resulting oil-in-water emulsion was poured into 60 mL of 1% (w/v) PVA solution, and dichloromethane was allowed to evaporate under magnetic stirring at room temperature overnight. The particles were collected at 20,000 × *g* for 10 min at 4 °C and washed thrice and subsequently lyophilised. For nanoparticles with a target size of 600 nm, 150 mg PLGA and 0.5% (w/v) PVA solution (Mw: 88–97 kDa; 87%–89% hydrolysed; Alfa Aesar) were used, and the particles were collected using centrifugation at 9500 × *g*. For 900-nm nanoparticles, 300 mg PLGA-NH_2_ and 1.2% (w/v) PVA solution (Mw: 88–97 kDa; 87–89% hydrolysed; Alfa Aesar) were collected via centrifugation at 7000 × *g*. All subsequent synthesis procedures were conducted as explained for the 300-nm nanoparticles. Fluorescent (APC) nanoparticles were obtained using an amine-reactive dye. Briefly, 1 mg of nanoparticles in 500 µL of distilled water was dispersed via water bath sonication for 5 min. The fluorescent dye was added at a concentration of 10 µg/mL and allowed to react for 20 min. The mixture was then washed thrice with PBS at 3500 rpm using a microcentrifuge and thoroughly dispersed. Particle size, polydispersity index (PDI), and zeta potential of the PLGA-NH_2_ nanoparticles were measured in triplicate using Zetasizer ZS3100 (Malvern Instruments Ltd., Worcestershire, UK).

### In vitro* cellular uptake study*

Mouse macrophage RAW264.7 cells (ATCC, USA) were seeded in 24-well plates at a density of 4 × 10^5^ cells/well for fluorescence microscopy. RAW264.7 cells were cultured in 500 µL of Dulbecco’s modified Eagle medium (Welgene Inc., South Korea) containing 10% foetal bovine serum (Gibco, Thermo Fisher Scientific) and 100 U/mL penicillin–streptomycin (Gibco) at 37 °C in a 5% CO_2_ incubator overnight. The cells were exposed to fluorescent PLGA nanoparticles at a final concentration of 200 µg/mL for 24 h, and latruculin A (1 µM), a phagocytosis inhibitor, was added 30 min before nanoparticle exposure. After incubation, the cells were washed thrice with phosphate-buffered saline (PBS). To visualise the intracellular uptake of nanoparticles, RAW264.7 cells were pipetted onto a slide and immersed in a drop of anti-fade solution. Optical channels were configured for DAPI (cell nuclei) and allophycocyanin (APC; nanoparticles). Intracellular particles were quantified based on their fluorescence intensity in randomly selected regions using ImageJ v1.52a (http://imagej.nih.gov/ij/). Mean fluorescence intensity of the internalised nanoparticles was normalised by dividing the total fluorescence intensity in selected regions by the total number of cells.

### ^***89***^***Zr labelling of PLGA-NH***_***2***_*** nanoparticles***

The conjugation of p-SCN-Bn-deferoxamine (DFO) to PLGA-NH_2_ nanoparticles and radiolabelling with ^89^Zr-oxalate (Korea Institute of Radiological and Medical Sciences, Seoul, Korea) was performed as previously described (Poot et al. 2019). Briefly, 5 mg of the synthesised PLGA-NH_2_ nanoparticles was thoroughly dispersed in 500 mL of PBS. Subsequently, 100 µg of amine-reactive p-SCN-Bn-deferoxamine (Macrocyclics, TX, USA) in dimethyl sulfoxide (2 mg/mL) was added to the nanoparticles, thoroughly mixed, and the pH was adjusted to 9.0 using 1.0 M Na_2_CO_3_ solution. The thiocarbamide bond reaction was allowed to proceed at 37 °C for 1 h at 550 rpm using a thermomixer. The DFO-conjugated PLGA-NH_2_ nanoparticles were collected via centrifugation at 3500 rpm for 5 min and washed thrice with PBS to remove unreacted p-SCN-Bn-deferoxamine. For ^89^Zr radiolabelling, 35–40 MBq of ^89^Zr-oxalate was diluted in 200 µL of 4-(2-hydroxyethyl)-1-piperazineethanesulfonic acid buffer (0.5 M), neutralised with 1.0 M Na_2_CO_3_. The ^89^Zr solution was mixed with the DFO-conjugated nanoparticles and radiolabelled at room temperature for 1 h at 350 rpm. The radiolabelled PLGA-NH_2_ nanoparticles were washed thrice with PBS, and the radiochemical yield was determined using a Bioscan AR-2000 radio-thin layer chromatography scanner (Eckert & Ziegler, Berlin, Germany).

### *Mouse model preparation for *in vivo* observation*

Twenty female BALB/c mice (6 weeks old or weight = 17.91 ± 0.38 g) were obtained from Jung-Ang Laboratory Animals (Seoul, South Korea). Intravenous administration was performed via the tail vein with an injection volume of 200 µL. Intranasal administration was performed after anaesthesia was induced via combined intraperitoneal ketamine (50 mg/kg) and xylazine (5 mg/kg) administration. The posture of the animal was maintained to ensure that the airway was in a horizontal position, and using a pipette, 10 µL of radio-labeled nanoparticles was dropped into the nostrils every 1 min, for a total of 40 µL, allowing for natural inhalation. Intramuscular administration was performed through the caudal thigh using a 60-µL injection volume, and subcutaneous administration was conducted via the subcutaneous tissue over the neck at a volume of 100 µL. Intranasal administration was performed with a pipette, whereas all other administrations were executed using a 30-G syringe needle. For each group, five mice were housed in a single cage.

### PET/CT and quantification

For PET imaging, ^89^Zr-PLGA-NH_2_ nanoparticles were injected through various routes (intravenous: 3.02 ± 0.33 MBq/mg; intranasal: 2.62 ± 0.24 MBq/mg; subcutaneous: 3.1 ± 0.14 MBq/mg; intramuscular: 2.92 ± 0.17 MBq/mg). The mice were placed on a platform and kept warm using a heating mat. PET/CT imaging was performed with the mice under 2% isoflurane anaesthesia using a Nanoscan PET/CT system (Mediso Ltd., Hungary). A static PET scan (10 min) and CT scan (480 projections, 50 kVp, 660 µA, 300 ms) were performed at 1, 6, 12, 24, 48, 72, 120, and 144 h after ^89^Zr-PLGA nanoparticle administration. The PET images were reconstructed using the Tera.gov reconstruction algorithm. Regions of interest were drawn over the lungs and main organs, and the uptake levels within the selected areas were quantified as the percentage of injected dose per gram of tissue (%ID/g), using Siemens Inveon Research Workplace (IRW2.0.0.1050).

### Fluorescence-activated cell sorting analysis

The mice were sacrificed 1, 6, 12, and 48 h following intranasal administration of APC-labelled PLGA nanoparticles (three mice at each time point). Cells were obtained from the lungs using a tumour dissociation kit (Miltenyi Biotech, Germany) according to the manufacturer’s instructions. The lungs were dissected and transferred into gentleMACS™ C tubes (Miltenyi Biotech, Bergisch-Gladbach, Germany) with an enzyme mix (2.35 mL RPMI 1640, 100 µL enzyme D, 50 µL enzyme R, and 12.5 µL enzyme A). The lung tissues were dissociated using a gentleMACS™ dissociator (Miltenyi Biotech). The samples were incubated at 37 °C with 5% CO_2_ under continuous rotation for 40 min. Red blood cells were collected using a 70-μm cell strainer and subsequently lysed for 5 min. To exclude dead cells, the lung cells were stained with Fixable Viability Stain 575 V (BD Biosciences, San Jose, CA, USA) for 20 min at room temperature away from light. The cells were washed with 2% foetal bovine serum in PBS. Subsequently, the cells were subjected to surface staining for 30 min at 4 °C, using the following antibodies: CD11c (BV510; BioLegend), CD64 CD11c (PE-CD594; BioLegend), Ly-6C (Gr-1) (BV421; BioLegend), CD170 (Siglec-F) (FITC; BioLegend), I-A/I-E(MHCII) (AF700; BioLegend), CD45 (APC-Cy7; BD Biosciences), and CD11b (PE-Cy7; BD Biosciences). The cells were then washed with 2% foetal bovine serum in PBS and fixed with cytofix/cytoperm solution for 20 min at 4 °C. The fixed cells were washed with perm/wash buffer (BD Biosciences) and stained with CD68 (PerCP/Cyanine5.5; BioLegend) antibody for 30 min at 4 °C. Fluorescence imaging and cell counting were conducted on a BD LSR Fortessa flow cytometer (BD Biosciences, USA); FlowJo v10.7.1 software was used for the analysis.

### Statistical analysis

All statistical analyses were performed using a two-tailed Student’s *t*-test. All results are expressed as mean ± standard error of the mean.

## Results

### ***Preparation and characterisation of PLGA-NH***_***2***_*** and fluorescently labelled nanoparticles***

**Fig. 1:**
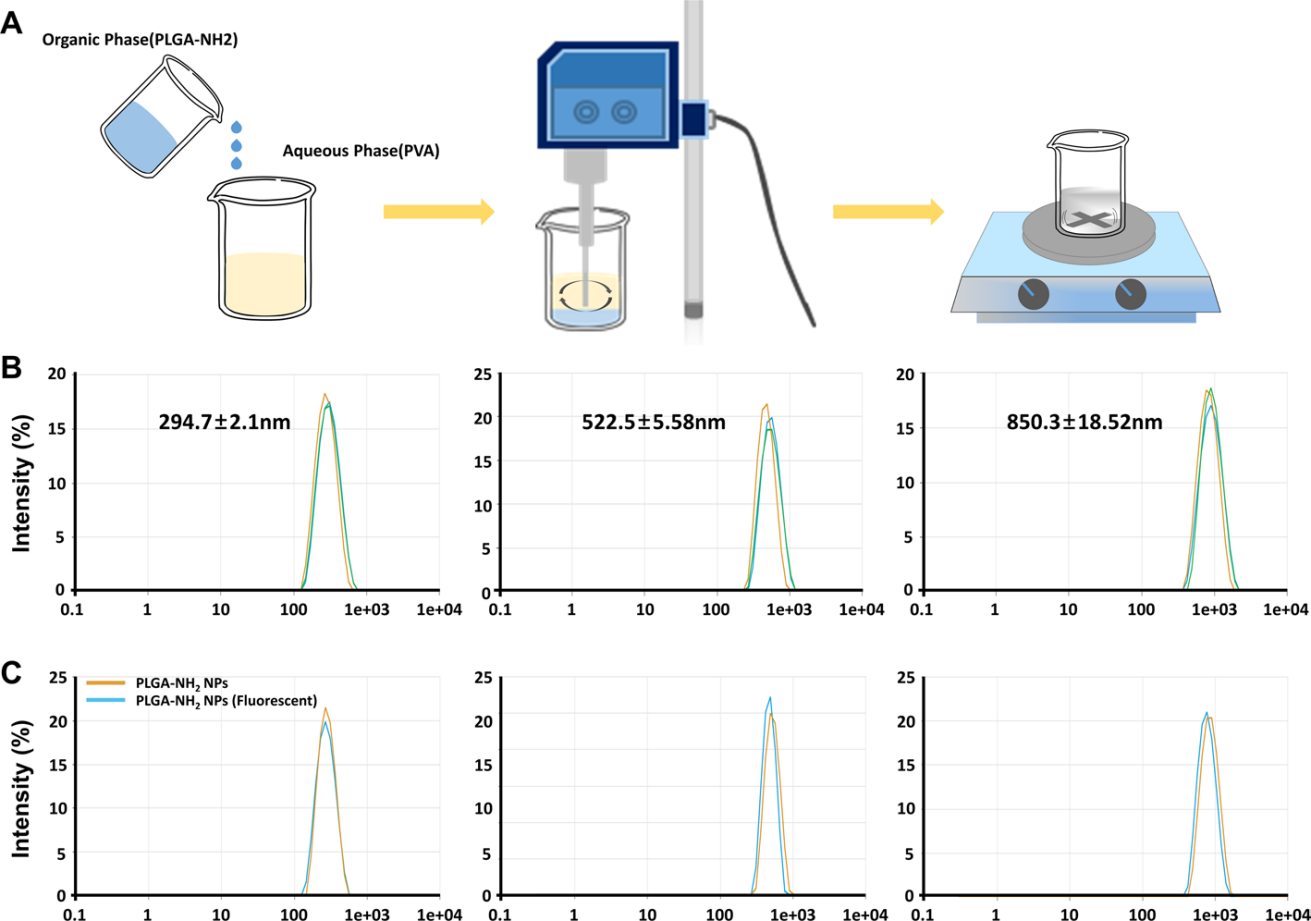
Preparation and characterisation of PLGA-NH_2_ nanoparticles. **a** Illustration of the oil-in-water single-emulsion solvent evaporation method. **b** Dynamic light scattering results displaying the sizes (small, medium, and large) of synthesised PLGA-NH_2_ nanoparticles. **c** Comparison of size between unlabelled and fluorophore-labelled nanoparticles. PLGA: poly(lactic-*co*-glycolic acid); NP: nanoparticle

PLGA-NH_2_ nanoparticles of three distinct sizes were synthesised by adjusting the w/v ratio (%) of PLGA-NH_2_, w/v ratio of PVA, and duration of sonication (Fig. 1a). The final characteristics of the synthesised nanoparticles, including their size, PDI, and zeta potential, are summarised in Table 1. Regarding particle dimensions, the smaller nanoparticles (NP-S) measured 294 ± 2.1 nm, intermediate-sized nanoparticles (NP-M) measured 522.5 ± 5.58 nm, and larger nanoparticles (NP-L) were 850 ± 18.52 nm in size (Fig. 1b). Fluorescently labelled nanoparticles (NP-F) displayed similar sizes, with NP-FS at 273.3 ± 3.02 nm, NP-FM at 572.7 ± 7.2 nm, and NP-FL at 824.5 ± 32.83 nm, indicating that the inclusion of the fluorescence tag did not substantially affect the particle size (Fig. 1c). Regarding zeta potential, all nanoparticles displayed similar negative charges—PLGA-NH_2_-S, -11.49 ± 3.46 mV; PLGA-NH_2_-M, − 11.74 ± 0.64 mV; and PLGA-NH_2_-L, − 9.57 ± 0.18 mV. The PDI values were 0.16 ± 0.06, 0.12 ± 0.11, and 0.12 ± 0.04, respectively, indicating homogenous size distribution of all nanoparticles.

**Table 1:** Physical characteristics of PLGA-NH_2_ nanoparticles of different sizes

Samples	Size (nm)	PDI	Zeta potential (mV)
PLGA_NH_2_-S	294 ± 2.1	0.16 ± 0.06	− 11.49 ± 3.46
PLGA_NH_2_-M	522.5 ± 5.58	0.12 ± 0.11	− 11.74 ± 0.64
PLGA_NH_2_-L	850 ± 18.52	0.12 ± 0.04	− 9.57 ± 0.18

*PDI* Polydispersity index; *PLGA* Poly(lactic-*co*-glycolic acid)

### Influence of nanoparticle size on cellular uptake in vitro

**Fig. 2:**
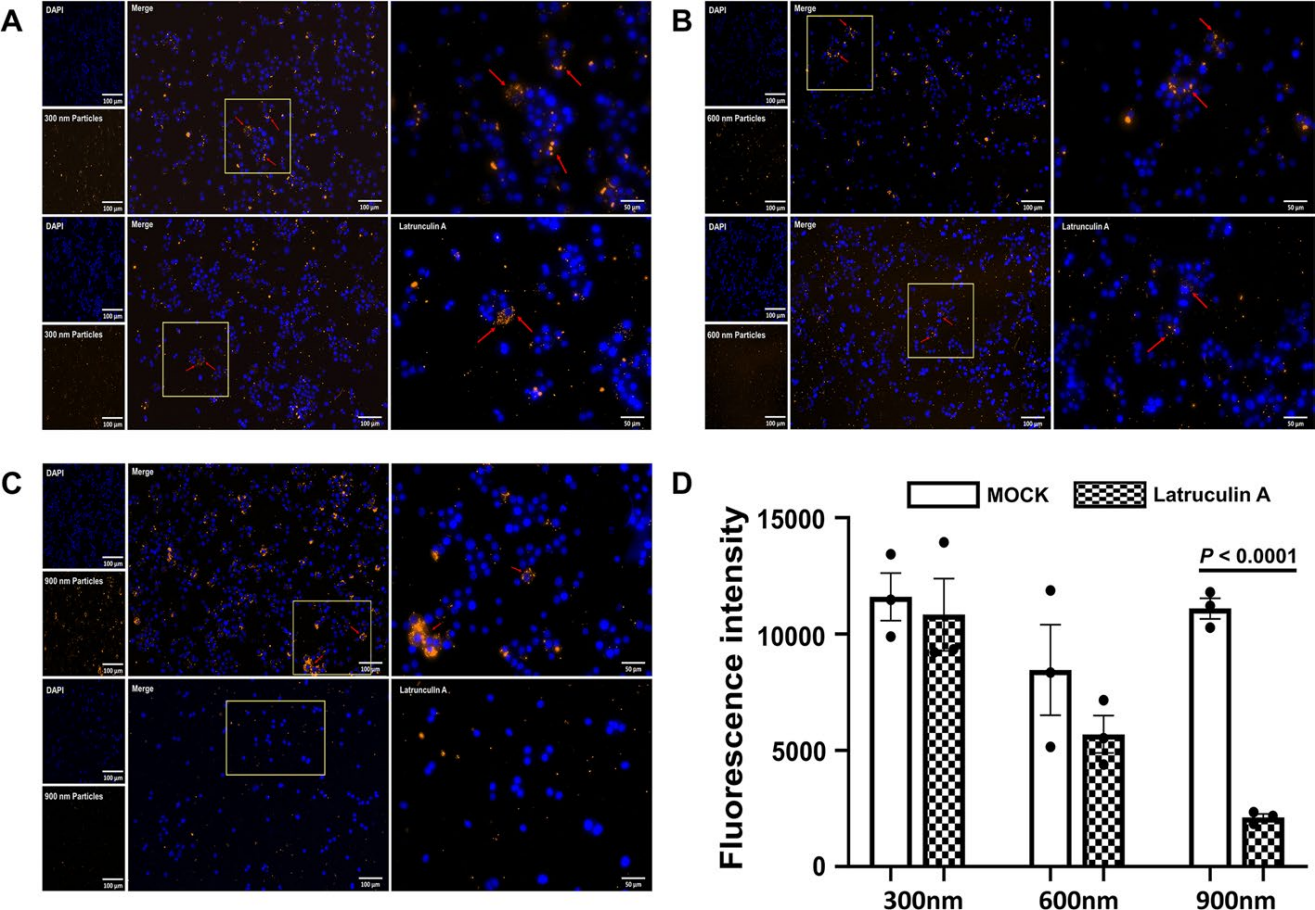
Microscopic examination of uptake of fluorescently labelled PLGA-NH_2_ nanoparticles by RAW264.7 cells after 24 h. Uptake of **a** small (300 nm), **b** intermediate (600 nm), and **c** large (900 nm) nanoparticles, following inhibition of phagocytosis. **d** Quantification plots depicting the uptake of nanoparticles of different sizes. Data are presented as mean ± SEM (n = 3). NP-F S/M/L: fluorescently labelled nanoparticles small/intermediate/large; PLGA: poly(lactic-*co*-glycolic acid); SEM: standard error of mean

We assessed the variations in the uptake of APC-labelled PLGA-NH_2_ nanoparticles by RAW264.7 cells based on size following a 24-h incubation period (Fig. 2). Additionally, we evaluated whether uptake was dependent on phagocytosis. After 24 h, RAW264.7 cells exhibited a preference for 300- and 900-nm nanoparticles, whereas the uptake of 600-nm nanoparticles was lower than that of the other nanoparticles. Upon inhibition of phagocytosis, the internalisation of 300-nm nanoparticles was minimally affected, whereas the internalisation of 900-nm nanoparticles decreased by 5.23-fold compared with that of the values before inhibition.

### ***Biodistribution of ***^***89***^***Zr-labelled PLGA-NH***_***2***_*** nanoparticles in mice***

**Fig. 3:**
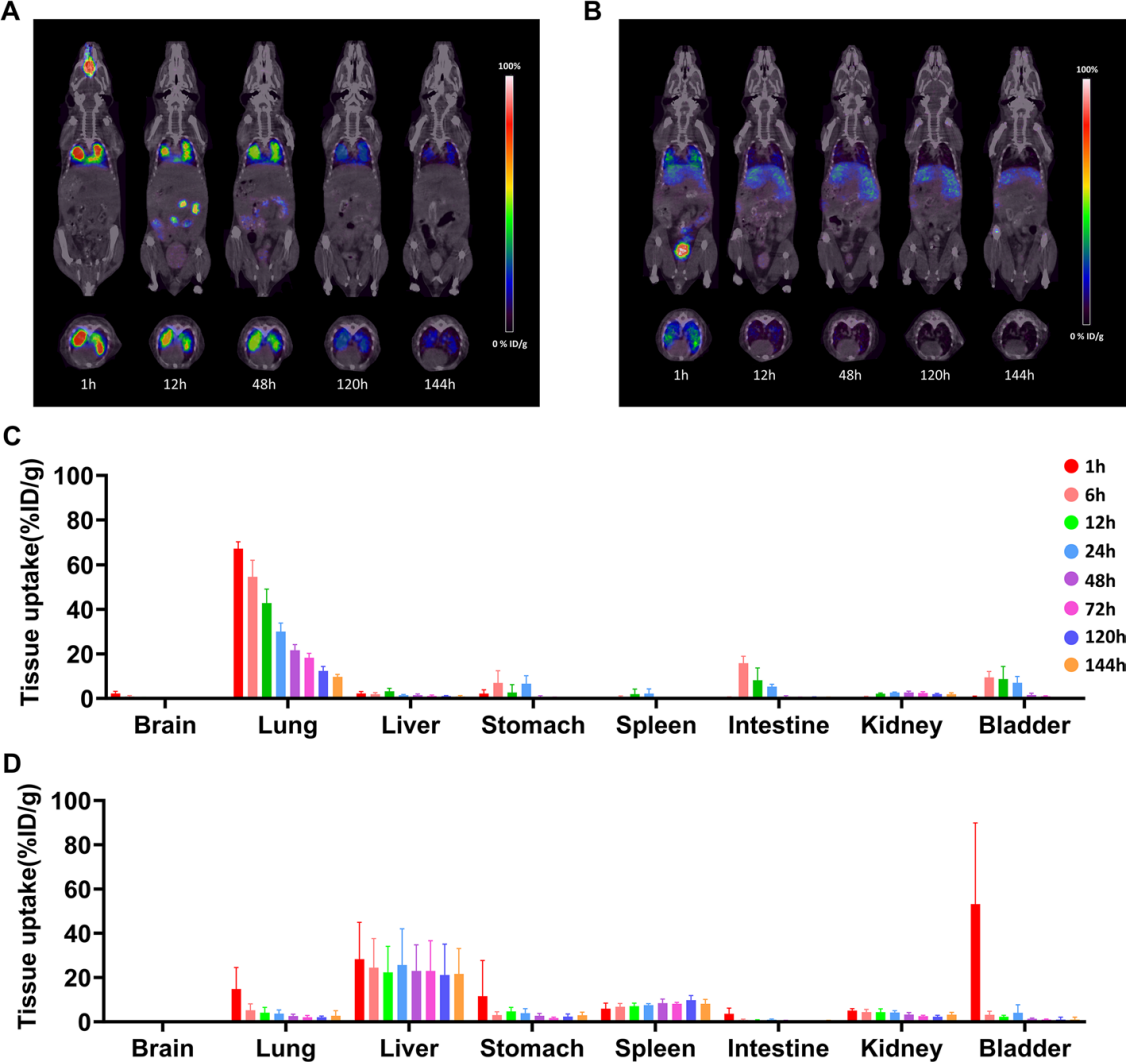
Biodistribution of ^89^Zr-PLGA-NH_2_ nanoparticles (900 nm) in mice. Representative coronal and axial PET images at 1, 12, 48, 120, and 144 h after **a** intravenous and **b** intranasal nanoparticle administrations. Nanoparticle uptake by major organs presented as %ID/g at 1, 6, 12, 24, 48, 72, 120, and 144 h following **c** intravenous and **d** intranasal administrations. Data are presented as mean ± SEM (n = 5). ^89^Zr-PLGA: radiolabelled poly(lactic-*co*-glycolic acid); ID: injected dose; PET: positron emission tomography; SEM: standard error of mean

To investigate the long-term systemic distribution, trafficking, and elimination dynamics of the nanoparticles in vivo, we used ^89^Zr, which has a half-life of 3.2 days, for PET imaging. The synthesised ^89^Zr-labelled PLGA-NH_2_ nanoparticles displayed a size of 885.5 ± 31.85 nm, charge of -14.55 ± 0.3 mV, and PDI of 0.362, with a radiochemical yield of 91.57%. Detailed information regarding the radiolabelling efficiency of PLGA-NH_2_ is provided in Additional file 1. In mice that were intravenously administered nanoparticles, we observed substantial distribution of the nanoparticles in the liver (28.3 ± 13.61%ID/g), lungs (14.76 ± 0.8%ID/g), and spleen (5.96 ± 2.04%ID/g). The initial distribution of nanoparticles in the liver remained consistent over 7 days, whereas that in the spleen gradually increased (Fig. 3a and 3c). In contrast, the distribution of nanoparticles in the lungs exhibited a sharp reduction after 1 h of administration, resulting in a 5.39-fold decrease relative to the initial distribution by day 7. In the case of nasal administration, a persistent distribution of nanoparticles in the lungs was observed throughout the study period (Fig. 3b and 3d). The lung distribution of nanoparticles was substantially higher (IN/IV %ID/g) than that in other major organs (1 h: 4.56-fold, 6 h: 10.42-fold, 12 h: 10.45-fold, 72 h: 8.89-fold, and 144 h: 3.54-fold) compared with that in the case of intravenous administration. Furthermore, nanoparticle accumulation in the liver and spleen of mice in the nasal administration group was almost negligible compared with that among mice in the intravenous administration group.

### Quantification of nanoparticle cellular uptake in the lung tissues

**Fig. 4:**
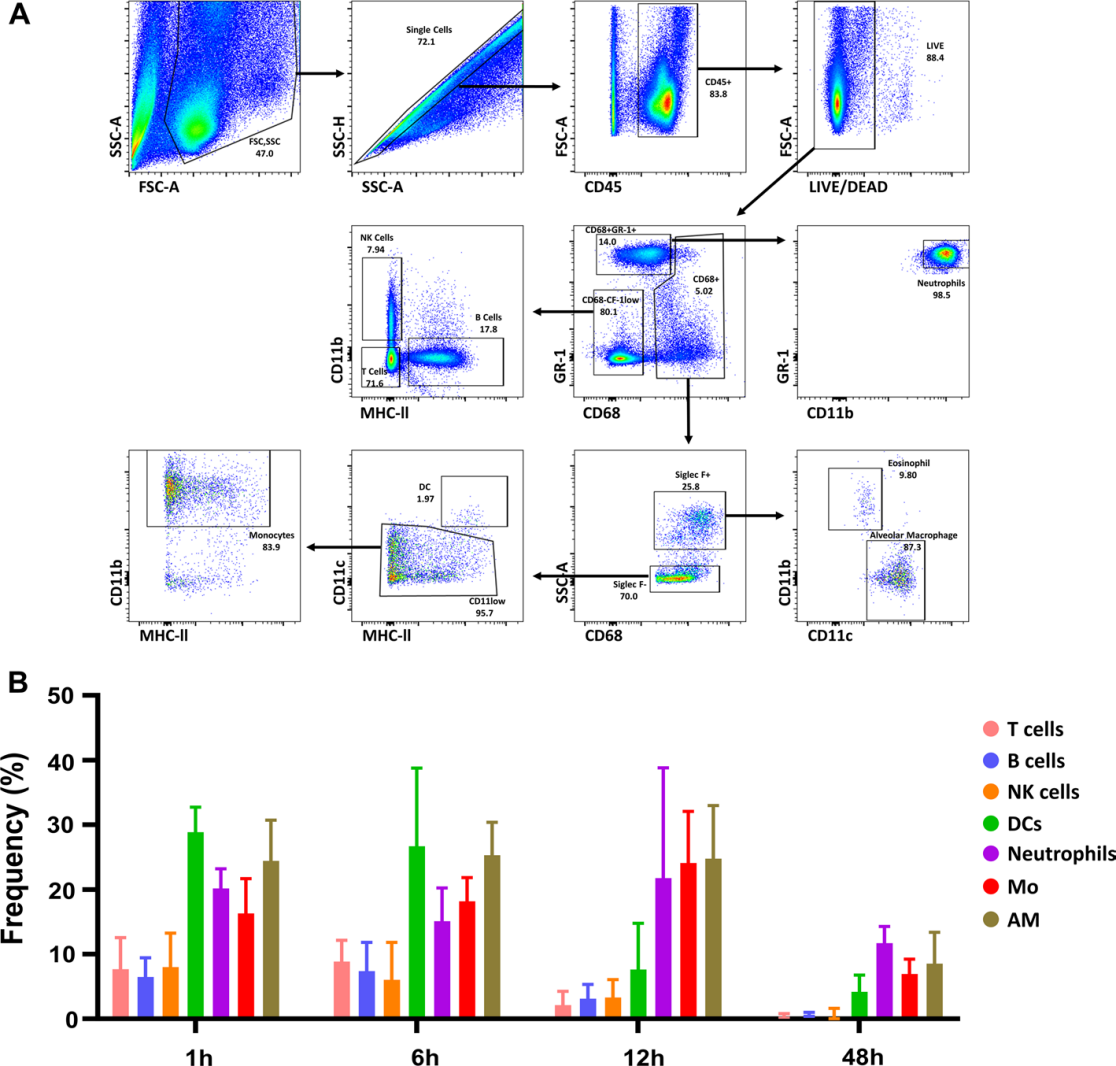
Preferential uptake of PLGA-NH_2_ nanoparticles (900 nm) by lung immune cells. **a** Gating strategy for the identification of lung immune cells. **b** Analysis of nanoparticle uptake by lung-resident immune cells at 1, 6, 12, and 48 h after intranasal administration. Data are presented as mean ± SEM (n = 3). PLGA: poly(lactic-*co*-glycolic acid)

We quantitatively analysed nanoparticle-positive immune cells using flow cytometry at 1, 6, 12, and 48 h after administration (Fig. 4a). Across all observed time points, immune cell subsets, including alveolar macrophages, dendritic cells (DCs), monocytes, and neutrophils, consistently exhibited a trend toward increased nanoparticle uptake compared to lymphoid immune cells such as T, B, and natural killer cells. Myeloid immune cells consistently demonstrated a relatively higher tendency for nanoparticle uptake than lymphoid immune cells, irrespective of the time after nanoparticle administration (Fig. 4b). Notably, most myeloid immune cells maintained elevated uptake levels for up to 6 h after nanoparticle administration, followed by a significant reduction at 48 h. DCs exhibited a sharp decline in nanoparticle uptake at 12 h, with the lowest uptake level among myeloid immune cells at 48 h.

## Discussion

In this study, we demonstrated a simple method for achieving efficient nanoparticle delivery to specific immune cells by adjusting particle size, as supported by both in vitro and in vivo experiment results. Specifically, we demonstrated the effectiveness of intranasal route in delivering PLGA nanoparticles to the lungs and achieving sustained retention primarily through engulfment by lung phagocytic cells. Additionally, drug dispersion to off-target organs was minimal. To the best of our knowledge, this is the first study to suggest the role of pulmonary immune cells in facilitating drug nanocarrier delivery and highlight the specificity of the delivery route within the context of nasal drug delivery (Haque et al. 2020; Liu et al. 2009).

The changes in size and zeta potential between the unlabelled and fluorescently labelled nanoparticles were negligible, consistent with other finding (Ma et al. 2021). Our results of internalisation experiments involving three submicron-sized nanoparticles and RAW264.7 murine macrophage cells suggested that the uptake of nanoparticles by phagocytic cells is size-dependent. Recent studies have reported similar findings of internalisation experiments involving macrophage cell lines and PLGA nanoparticles, emphasising that larger nanoparticles are more readily engulfed by phagocytic cells, even early after administration (Haque et al. 2020; Liu et al. 2022). Utilising nanoparticles smaller than 1000 nm can lead to a rapid delivery of biomolecules to tissue-resident phagocytic cells. The phagocytosis-inhibition assay revealed that smaller nanoparticles primarily rely on endocytosis for cellular uptake, whereas larger nanoparticles are mainly internalised through phagocytosis. By monitoring the distribution patterns of nanoparticles within immune cells, various applications can be developed for scenarios involving infections or inflammation caused by different pathogens. This approach facilitates targeted delivery to specific immune cells, while avoiding non-specific cellular uptake through endocytosis. Hence, the synthesis of uniformly sized nanoparticles is essential for this purpose.

In our in vivo experiments, radiolabelled nanoparticles exhibited significantly higher pulmonary uptake when administered intranasally rather than intravenously. Consistent with previous study findings, intravenously administered nanoparticles demonstrated substantial distribution in filter organs such as the liver and spleen, with a rapid 2.8-fold decrease in lung delivery within 6 h, suggesting that it is an inefficient route for pulmonary drug delivery (Mitchell et al. 2021; Haque et al. 2020; Park et al. 2016). In contrast, nanoparticles delivered to the lungs via the nasal route exhibited a more gradual decrease over 7 days, indicating increased retention and the potential for sustained drug delivery to the tissue. Moreover, as the nasal route targets the lungs directly and avoids systemic circulation, we observed less ectopic delivery of nanoparticles to off-target organs. In the case of intramuscular and subcutaneous injections, most nanoparticles remained around the injection site throughout the tracking period, with only a small quantity being eliminated by the kidneys (Additional file 2). Therefore, these injection routes do not appear suitable for efficiently targeting the lungs.

Following intranasal administration, nanoparticle uptake by myeloid immune cells in the lungs consistently exceeded that of lymphoid immune cells at all examined time points. Moreover, internalisation by lymphoid immune cells initially increased but significantly decreased over time. These findings align with the observed trend that synthesised nanoparticles exhibit high phagocytic uptake by myeloid immune cells and relatively lower uptake by non-phagocytic cells (Liu et al. 2022). The findings suggests that when nanoparticles with similar physicochemical properties are administered to the lungs, their uptake by lymphoid immune cells is minimised, whereas drug delivery efficiency to myeloid immune cells increases and the negative charge of the nanoparticle is also one of the factors contributing to an increase in its uptake by tissue-resident immune cells (Xiao et al. 2011). DCs, which can originate from both myeloid and lymphoid cells, displayed initially high nanoparticle uptake followed by a sharp decline based on flow cytometry measurements. In contrast, in the PET analysis, spleen uptake increased up to 24 h post-administration. Considering these findings and the immunological behaviour of DCs as they migrate to secondary lymphoid organs to present antigens, Manolova et al. tracked 1000-nm nanoparticles injected into the footpad, demonstrating that these nanoparticles were predominantly observed within the medullary and paracortical regions of the lymph nodes, which are mainly enriched in DCs, rather than within B-cell follicles (Manolova et al. 2008). Therefore, nanoparticles administered intranasally may likely move from the lungs to the spleen within 12 h. The lung pulmonary macrophages maintained a relatively high uptake rate for up to 48 h. This finding indicates that local immunity can be selectively modulated by strategically selecting nanoparticles and their physicochemical characteristics. Furthermore, when administering vaccine compounds using nanoparticles, this strategy may be employed to effectively deliver antigens to secondary lymphoid organs adjacent to the nasal route, particularly 6 h after intranasal administration, as previously demonstrated.

Our study has some limitations. First, our research findings were confined to rodent models, and for translation to clinical settings, investigations must be expanded to larger animals, such as primates. This would entail replicating intranasal administration techniques suitable for large animals in clinical settings, thereby addressing any species-specific effects. Second, during intranasal administration, a small number of nanoparticles entered the stomach, as confirmed using PET imaging. Previous research has highlighted suboptimal optimisation of intranasal administration methods, resulting in the entry of nanoparticles to the stomach, which can lead to reduced lung-delivery efficiency compared to our results, even in the initial stages of administration (Wu et al. 2020).

## Conclusions

We demonstrated the size-dependent preferential uptake of nanoparticles by immune cells, along with the superior efficiency of drug delivery via the intranasal route when targeting the lung tissues. We also identified diverse immune cell uptake patterns. These findings highlight the potential of intranasal drug delivery for precise pulmonary targeting while minimising off-target effects on major organs. The findings demonstrate the viability of our NP-based drug delivery strategy for treating diseases associated with myeloid cells within the lungs, offering avenues for symptom relief and effective delivery of vaccine candidates. Our study provides valuable information for the advancement and practical applications of PLGA-based nanocarrier systems designed for precise drug delivery, with potential applications in addressing lung diseases, such as COVID-19, and supporting further vaccine development.

### Supplementary Information

**Additional file 1.** Radiolabelling efficiency. Radio-TLC analysis of ^89^Zr-PLGA-NH2 nanoparticles (A, B, C, and D) after 1 h and (E) after washing with PBS. ^89^Zr-PLGA: radiolabelled poly(lactic-*co*-glycolic acid); PBS: phosphate-buffered saline; TLC: thin-layer chromatography.

**Additional file 2.** Biodistribution of ^89^Zr-PLGA-NH2 nanoparticles in major tissues following A ntramuscular and B subcutaneous injections in mice. ^89^Zr-PLGA: radiolabelled poly (lactic-co-glycolic acid).

## Data Availability

The datasets generated and analysed in the current study are available from the corresponding author on reasonable request.

## Abbreviations

APC: Allophycocyanin
DFO: Deferoxamine
DC: Dendritic cell
NP-F: Fluorescently labelled nanoparticles
NP-M: Intermediate-sized nanoparticles
NP-L: Larger nanoparticles
MRI: Magnetic resonance imaging
PBS: Phosphate-buffered saline
PLGA: Poly(lactic-*co*-glycolic acid)
PDI: Polydispersity index
PVA: Polyvinyl alcohol
PET: Positron emission tomography
NP-S: Smaller nanoparticles
