# Supplementary material for: Intranasal administration enhances size-dependent pulmonary phagocytic uptake of poly(lactic-co-glycolic acid) nanoparticles
Source: EJNMMI Radiopharm Chem. 2024 Feb 15;9:12. doi: 10.1186/s41181-023-00227-x (PMC10869321; doi:10.1186/s41181-023-00227-x)
Supplement: Supplementary file 1 — Additional file 1. Radiolabelling efficiency. Radio-TLC analysis of 89Zr-PLGA-NH2 nanoparticles (A, B, C, and D) after 1 h and (E) after washing with PBS. 89Zr-PLGA: radiolabelled poly(lactic-co-glycolic acid); PBS: phosphate-buffered saline; TLC: thin-layer chromatography. [file 41181_2023_227_MOESM1_ESM.docx]

**Additional File 1**

**
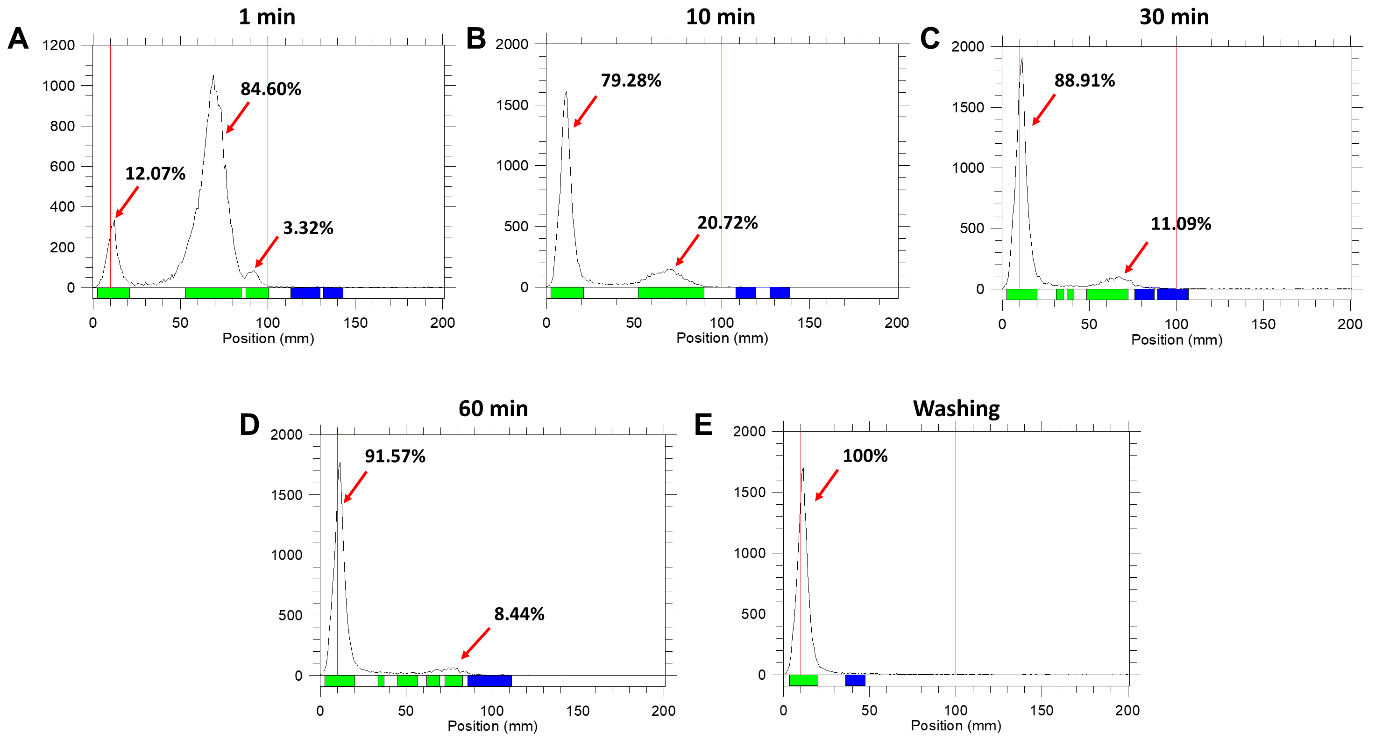
**

**Additional File 1.** Radiolabelling efficiency. Radio-TLC analysis of ^89^Zr-PLGA-NH_2_ nanoparticles (a, b, c, and d) after 1 h and (e) after washing with PBS.

^89^Zr-PLGA: radiolabelled poly (lactic-co-glycolic acid); PBS: phosphate-buffered saline; TLC: thin-layer chromatography
