# Supplementary material for: Intranasal administration enhances size-dependent pulmonary phagocytic uptake of poly(lactic-co-glycolic acid) nanoparticles
Source: EJNMMI Radiopharm Chem. 2024 Feb 15;9:12. doi: 10.1186/s41181-023-00227-x (PMC10869321; doi:10.1186/s41181-023-00227-x)
Supplement: Supplementary file 2 — Additional file 2. Biodistribution of 89Zr-PLGA-NH2 nanoparticles in major tissues following A ntramuscular and B subcutaneous injections in mice. 89Zr-PLGA: radiolabelled poly (lactic-co-glycolic acid). [file 41181_2023_227_MOESM2_ESM.docx]

**Additional File 2**

**
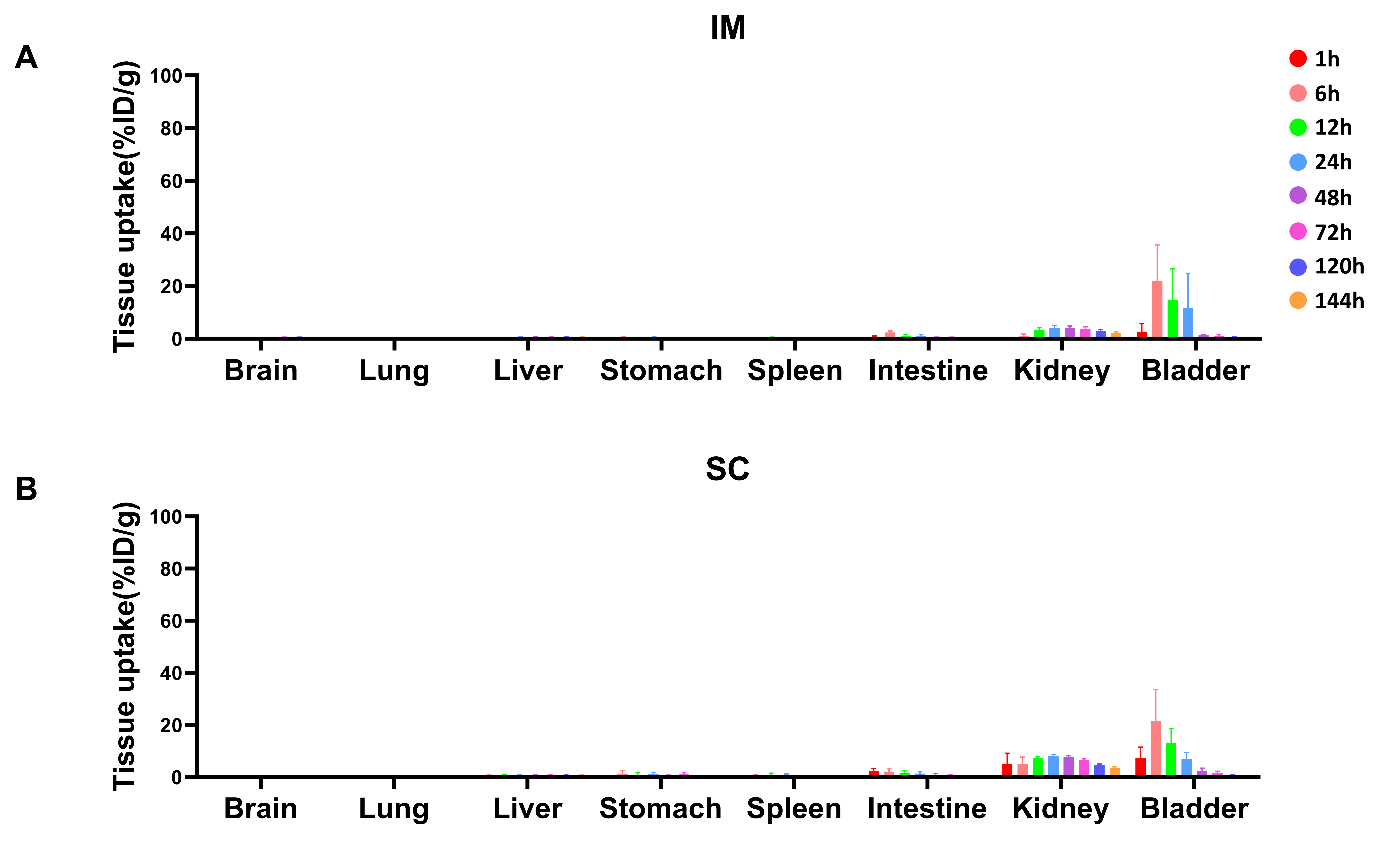
**

**Additional File 2.** Biodistribution of ^89^Zr-PLGA-NH_2_ nanoparticles in major tissues following (a) intramuscular and (b) subcutaneous injections in mice.

^89^Zr-PLGA: radiolabelled poly (lactic-co-glycolic acid)
